# Design and Evaluation of Low-Cost Vibration-Based Machine Monitoring System for Hay Rotary Tedder

**DOI:** 10.3390/s22114072

**Published:** 2022-05-27

**Authors:** Arkadiusz Mystkowski, Rafał Kociszewski, Adam Kotowski, Maciej Ciężkowski, Wojciech Wojtkowski, Michał Ostaszewski, Zbigniew Kulesza, Adam Wolniakowski, Grzegorz Kraszewski, Adam Idzkowski

**Affiliations:** Faculty of Electrical Engineering, Bialystok University of Technology, Wiejska St. 45D, 15351 Bialystok, Poland; a.mystkowski@pb.edu.pl (A.M.); r.kociszewski@pb.edu.pl (R.K.); a.kotowski@pb.edu.pl (A.K.); m.ciezkowski@pb.edu.pl (M.C.); w.wojtkowski@pb.edu.pl (W.W.); m.ostaszewski@pb.edu.pl (M.O.); z.kulesza@pb.edu.pl (Z.K.); a.wolniakowski@pb.edu.pl (A.W.); g.kraszewski@pb.edu.pl (G.K.)

**Keywords:** accelerometer, vibration monitoring, machine health monitoring, low-cost sensors, agriculture machinery

## Abstract

Vibration monitoring provides a good-quality source of information about the health condition of machines, and it is often based on the use of accelerometers. This article focuses on the use of accelerometer sensors in fabricating a low-cost system for monitoring vibrations in agricultural machines, such as rotary tedders. The aim of the study is to provide useful data on equipment health for improving the durability of such machinery. The electronic prototype, based on the low-cost AVR microcontroller ATmega128 with 10-bit ADC performing a 12-bit measurement, is able to acquire data from an accelerometer weighing up to 10 g. Three sensors were exposed to low accelerations with the use of an exciter, and their static characteristics were presented. Standard experimental tests were used to evaluate the constructed machine monitoring system. The self-contained prototype system was calibrated in a laboratory test rig, and sinusoidal and multisinusoidal excitations were used. Measurements in time and frequency domains were carried out. The amplitude characteristic of the preformed system differed by no more than 15% within a frequency range of 10 Hz–10 kHz, compared to the AVM4000 commercial product. Finally, the system was experimentally tested to measure acceleration at three characteristic points in a rotational tedder, i.e., the solid grease gearbox, the drive shaft bearing and the main frame. The RMS amplitude values of the shaft vibrations on the bearing in relation to the change in the drive shaft speed of two tedders of the same type were evaluated and compared. Additionally, the parameters of kurtosis and crest factor were compared to ascertain the bearing condition.

## 1. Introduction

Mechanical vibration transducers are used to receive a mechanical signal from a vibrating object and convert it into an electrical signal. This signal is properly processed. A measurement system includes a vibration transducer, for instance, a piezoelectric transducer, and an appropriate amplifier. Mechanical vibration measurement systems measure the vibrations produced by machines [1] as well as the vibrations of buildings or bridges [2]. Improving the performance of tool condition monitoring (TCM) systems is one of the challenges still facing manufacturers [3].

Nowadays, manufacturers of agriculture and farming equipment are interested in machine monitoring systems, which check if a machine is productive, or in condition monitoring systems, which have the ability to assess the health of a machine over a period of time. This helps in minimizing wear and tear in parts such as bearings and gearboxes, reducing unplanned machine downtime and enabling automation based on real-time machine condition data [4]. A rotary tedder that includes multiple rotor gearboxes is an example of a machine incorporating a condition monitoring system.

Accelerometric sensors for measuring vibration parameters are divided into the piezoelectric [5] and MEMS (micro electro-mechanical systems) categories. The latter can be piezoresistive [6] or differential capacitive [7]. The choice of sensor depends on the application. Piezoelectric sensors are not suitable for static measurements, i.e., gravity g and overload tests, such as multiple-g accelerations caused by the thrust of a spacecraft taking off, or airplane maneuvers. Unlike capacitive and piezoresistive sensors, they are more suitable for seismic measurements where frequencies are less than 1 Hz (earthquakes, vibrations of buildings, bridges). The operating frequency range of these sensors is wide, and their use also includes measuring vibrations of electric machines, detecting faults in gears, and monitoring the operation of wind turbines. Piezoelectric sensors must be characterized by high sensitivity and a low level of transverse vibrations, and the operating frequency range should be lower than the resonance frequency [8].

Until now, there has been limited research into low-cost devices that could help to track machine efficiency or to enhance the durability of agricultural machine components. The majority of research on vibration in agriculture refers to user comfort (the exposure to vibration) and safety [9].

## 2. Literature Review

### 2.1. High-Performance and Low-Cost Sensing

In the publication [10], the authors compare the parameters of the accelerometric sensors commonly found in the literature. These sensors range from the most expensive on the market (380–2070 €) to the cheapest (5.4–12 €). They often have similar ranges of ±2 g (3713B112G—triaxial MEMS DC response accelerometer, PCB Piezotronics) or ±3.6 g (ADXL335—triaxial low-power accelerometer, Analog Devices) and similar low operating frequencies of 0–250 Hz (3713B112G), 0.5–550 Hz (ADXL335). In contrast, cheaper differential capacitive sensors, such as ADXL335, usually have a higher spectral noise density of 300 µg/√Hz compared to the more expensive 3713B112G (22.9 µg/√Hz). Piezoelectric single-axis sensors KS48C (0.6 µg/√Hz) or KB12VD (0.06 µg/√Hz) are characterized by the lowest values of spectral noise density.

Other important parameters are the resolution (sensitivity) of the acceleration measurement, and the signal-to-noise ratio. The authors of the publication [11] measured the vibrations of wind turbines with a wireless system based on ADXL355 sensors (sensitivity 3.9 µg/LSB, noise spectral density 22.5 µg/√Hz) and compared the measurement results with the 3713B112G sensors in the registered stationary system (0.25 mg rms, 22.9 µg/√Hz). They observed that the sensor resolution of 1 mg may not be sufficient for the analysis of vibrations of wind turbine poles, and that it prevents analysis of higher frequencies with a good signal-to-noise ratio. The noise of the ADXL355 sensor depends on the sampling frequency. The measured mean standard deviation of the noise is 0.44 mg (1000 Hz), 0.19 mg (100 Hz) and 0.056 mg (15 Hz) [12].

A low-cost data-acquisition system based on Raspberry-Pi with a high sampling frequency (50 kHz) and a recording capacity of up to three channels, is proposed in publication [13]. It is designed for monitoring bearings and determining bearing faults where signals are processed by frequency methods (FFT, STFT, wavelets) [14]. Generally, the cost of equipment for registering vibrations is very high. It is 4000 EUR for the basic version of the AVM4000 (AMC VIBRO, Kraków, Poland) which is a multichannel device (4–24 channels). However, there are cheaper vibration monitoring devices available (one or two channels). The challenge for low-cost solutions is to use cheaper devices with an internal ADC of 10-bit or 12-bit, such as the ATmega 1280, PIC18F4550, USB NI-6008 with a sampling rate of 10–100 kHz and with an internal memory up to 512 bytes for buffering data [15]. If more functionalities of the DAQ unit are needed—for example, for wireless IoT condition monitoring—the use of an ESP32-WROVER SoC microcontroller unit with an integrated 2.4 GHz radio module, which enables Wi-Fi and Bluetooth connectivity, appears to be a good solution [16].

### 2.2. Vibration Calibration of Accelerometers

The instrument used to measure vibrations is a mechanical vibration calibrator. It is a standard source of vibrations consisting of a portable vibration exciter used to check the metrological properties of the measuring system. With this device, one can check whether the system correctly measures vibration parameters, such as amplitude (displacement), velocity or acceleration.

The standard vibration transducer used to transmit vibrations must have appropriate metrological properties and be very stable. The tested sensor and its wiring must be properly mounted to avoid erroneous results [17]. When calibrating, the ISO 16063-21 standard is used, in which the comparative method is described [18]. It consists in subjecting two transducers (the model transducer and the transducer of unknown sensitivity) to vibrations of the same values. The output signals from both transducers are measured. Knowing the sensitivity of the reference sensor, one can determine the parameters of the calibrated sensor.

The sensitivity of a transducer is defined as the ratio of the output signal, e.g., the voltage, to the input signal, e.g., the vibration acceleration to which the transducer is subjected. It provides a moderate accuracy (uncertainty) of measurement. The most accurate method is the absolute method, i.e., with reference to the basic SI units, such as meter, second, kilogram and electric current. This is considered the national standard. According to the ISO 16063-11 Standard, the absolute method is used to calibrate working standards or laboratory standards [19]. The sensitivity of such vibration transducers is determined over the entire operating frequency range (from 1 Hz to 10 kHz). A calibrated vibration transducer (sensor) is subjected to controlled sinusoidal vibrations, the values of which (amplitude and phase) are measured by the interferometric method. The laser beam is directed to the mounting surface and the voltage from the calibrated accelerometer is measured at the same time. Laser interferometers are very precise devices for measuring distance (displacement) with an accuracy of ± 100 nm in the range of 250 mm [7]. The measurement uncertainty is influenced by the instability of the frequency of the light source, as well as changes in environmental parameters, e.g., the temperature in the vicinity of the laser beam source.

### 2.3. Static and Dynamic Performance

Optical methods with the use of a QPD (four-quadrant photodetector) are also used to measure vibrations of low frequencies and low amplitudes. The optical accelerometer is characterized by high sensitivity (1.74 V/(ms^−2^), a linear conversion function in the range of 0.4 to 12 Hz, the range of measured accelerations from 0.003 to 7.29 m/s^2^ and a noise spectral density of 160 µms^−2^/√Hz [20].

The calibration of accelerometric sensors is divided into static and dynamic categories. The method of static and dynamic calibration of low-cost 3D accelerometric sensors in the frequency range of 0–10 Hz is presented in the publication [21]. The transducer can be calibrated with a single frequency signal or a frequency sweep, or a random noise function can be used. The publication [22] discusses the methods of dynamic calibration for various types of amplifiers with bridge, charge and voltage output. The method of calibration of charge amplifiers using multisinusoidal input is presented in article [23]. Seismic IEPE (Integrated Electronics Piezo-Electric) sensors with ultra-low noise operating in the frequency range from 0.003 Hz to 200 Hz cooperate with these types of amplifiers. The method of measuring sensor noise is presented in [24]. The publication [25] compares the parameters of the Meggitt model 731A, the PCB model 393B31, the Dytran model 3191A1 and the Colibrys model SF3000—all sensors used in earthquake prediction systems. The publication [26] presents three methods of calibration for piezoelectric sensors and MEMS, and [27] outlines a method for estimating the measurement uncertainty of accelerometer sensitivity.

### 2.4. Research Purpose

The literature review showed that few low-cost instruments are available to measure low accelerations with satisfactory accuracy by comparison with commercial hardware. This article is focused on designing and experimentally testing an electronic prototype for a machine monitoring system. The aim of this publication is to present the possibilities of using piezoelectric and MEMS sensors in the construction of a low-cost system for measuring vibrations in agricultural machines (tedders, rotary rakes). The machine with the implemented monitoring system is intended for harvesting large fields of crops as green feed, in accordance with the concept of the Agricultural Valley 4.0. The role of the system is to control the selected operating parameters and to assure self-diagnostic information on the wear of machine parts. The development was also focused on simplicity and user friendliness in configuring and operating the low-cost device, as well as in data collection and post-processing. The main proposals of our study are listed below:

- Using a low-cost AVR microcontroller for such an application. The unit does not need any operating system, and this makes programming easier than Raspberry Pi, for example. This significantly reduces time consumption for creating software and the total cost of the system. The majority of low-cost solutions use high-level programming languages. Our tests confirmed that computation time for a program expressed in an AVR assembler is 2–3 times faster than its equivalent in the C language.
- Blending self-created hardware and software for decision support. The software, which is based on statistical features, such as RMS, kurtosis and crest factors, enables the differentiation of the worn parts of a rotary tedder from fully-operational parts. Experimental tests showed that the electronic prototype ensures acceptable accuracy of acceleration measurement at low frequencies for low-acceleration amplitude when compared with the commercial system.

## 3. Materials and Methods

The simulation analysis of the proposed signal conditioning system, laboratory tests of three selected sensors (two piezoelectric and one MEMS) and the experimental verification of the electronic prototype on agricultural hay tedders in real conditions were the objects of this study. The FFT (fast Fourier transform) analysis was performed with the LTSpice simulator (Analog Devices, Wilmington, MA, USA).

To validate the performance in laboratory conditions, the constructed electronic prototype was compared with the commercial product (AVM4000, AMC VIBRO, Kraków, Poland) [28]. In this test, dynamic movements with low-range amplitudes and frequencies ranging from 10 Hz to 10 kHz were tested. The swept sine frequency response method was used (at a sample rate of 20 kHz and 400 spectral lines).

To validate the performance of the system in real conditions, the prototyping model was tested on agricultural machines (two rotary tedders P8-890, SaMASZ, Zabłudów, Poland) [29]. The first of these machines was produced in 2019 (a moderately used machine) and the second was produced in 2020 (a more frequently used machine that had worked over 600 hectares in the year 2021).

The tests relied on acquiring data from three general-purpose piezoelectric accelerometers for industrial applications, mounted at different characteristic points of the machine during hay tedding. These three points were: the first left gearbox of the tedder, the first left bearing of the PTO (power take-off) drive shaft and the central point of the main frame. The measuring positions were perpendicular to the shaft due to radial loads, and parallel to the gearbox due to axial loads.

Time-domain techniques were used to determine the wear rate of machine components. Using the time-domain signal, a defect can be detected and its magnitude assessed using statistics indicators, such as the energy content (Root Mean Square—RMS), crest factor (CF), kurtosis (KU) or energy index (EI) [30]. The sliding window method for framing a time-series dataset was used. The one- and ten-second-long subsets were analyzed and compared for both machines.

## 4. Simulation Analysis of the Signal Conditioner

**Figure 1:**
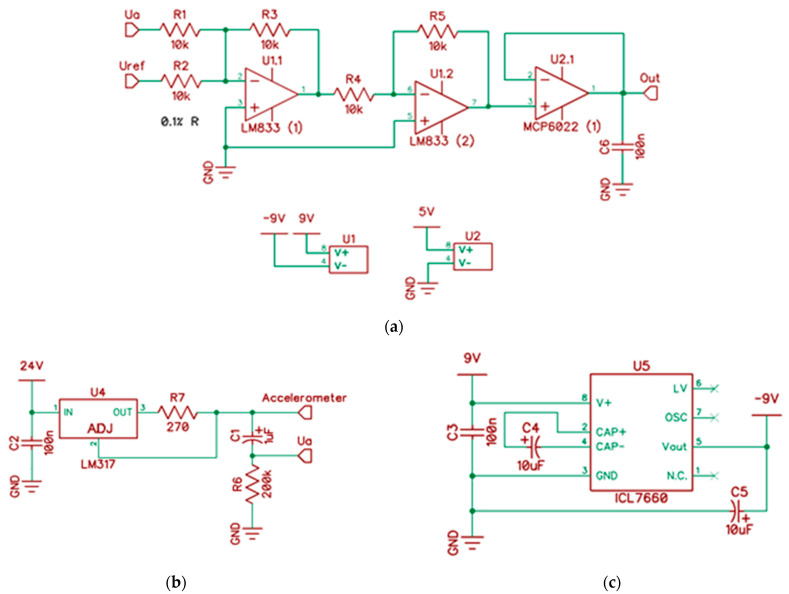
(**a**) Signal conditioning circuit (one channel) for measuring acceleration; (**b**) LM317 adjustable regulator as a constant current supply for IEPE accelerometer; (**c**) symmetric power supply for LM833 operational amplifiers.

Integrated Electronics Piezo-Electric (IEPE) sensors, i.e., the MTN/2200 series (Monitran Ltd., High Wycombe, UK), must be supplied by a constant current source. The signal from the accelerometer must be amplified. Figure 1 shows the summing amplifier U1.1 [31]. The voltage *V_ref_* = 2.5 V sets the signal level to which the voltage *V_a_* is added

(1) $$V_{01}=-\left(\frac{R_{3}}{R_{1}}V_{a}+\frac{R_{3}}{R_{2}}V_{ref}\right).$$

The U1.2 amplifier inverts the signal—the output is 180° out of phase to the input

(2) $$V_{02}=-\frac{R_{5}}{R_{4}}V_{01}.$$

The amplifier U2.1 with the gain *k_u_* = 1, which is supplied with a single voltage of 5 V, acts as a buffer protecting the input of the ADC converter of the microcontroller against exceeding the supply voltage, and against the voltage dropping below the GND level. As a consequence of this, it is possible to detect acceleration within the range of ±25 g.

In Figure 1b, the IEPE accelerometer is powered from the voltage source U4. The supply voltage recommended by the manufacturer should be within the range of 18–30 VDC [32]. In Figure 1c, the U5 unit is presented (ICL7660). It generates a symmetrical ±9 V power supply to the U1 (LM833) operational amplifiers.

**Figure 2:**
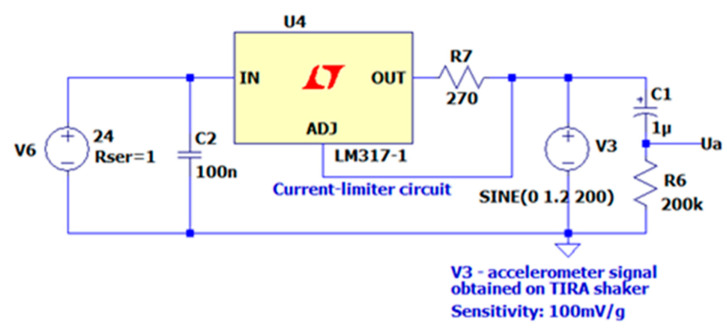
A circuit for supplying a piezoelectric accelerometer—LT Spice simulation.

The LM317 stabilizer operates as a constant current source (Figure 2). In this case, according to the application note, the current (I = 1.2/R7) is within the obligatory range of 0.5–8 mA for the accelerometers used. The V3 source reflects the accelerometer output during the tests on a vibration shaker, which was excited by a sinusoidal signal from an external signal generator. The amplitude at the level of 1.2 V at the sensitivity of 100 mV/g means the acceleration of 12 g. The capacitor C1 cuts the DC component. C2 is the required capacity for correct operation of U4.

### Spectral Analysis

To reduce the spectral leakage caused by finite-length sampling during frequency analysis, *N* signal samples are multiplied in the time domain with a given time window. In the data windowing method, the original signal *x*(*n*), where n∈{0.1.…N−1} is modified by multiplication with a windowing function that approaches zero near *n* = 0 and *n* = *N* − 1 and reaches a peak near *N*/2. There are a number of time windows; the Hamming windows is one of the most popular. If the window is not selected, the rectangular window is used as default. The Hamming window function *w*(*n*) is given below [33].

(3) $$w(n)=0.54-0.46\cos\left(\frac{2\pi n}{N-1}\right),\;0\leq n\leq N-1.$$

The signal is multiplied by a window function

(4) $$x_{0}(n)=x(n)\cdot w(n).$$

A decibel expression of spectral amplitude is given as

(5) $$X_{0}(j\omega_{m})=20\log_{10}\left|\sum_{n=0}^{N-1}x_{0}(n)\;e^{-jn\omega_{m}}\right|.$$

where $\omega_{m}$ is the frequency-determining component equal to 2πm/N, which is a normalized value.

**Figure 3:**
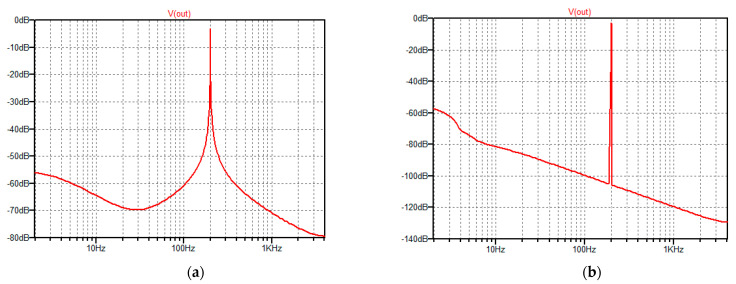
Spectral amplitude responses for the input acceleration of 10 g given on input *V*_a_ as the sine function with frequency of 200 Hz; simulation time equals 505.5 ms, *N* = 4096. (**a**) Rectangular window; (**b**) Hamming window.

In Figure 3a,b the spectral amplitudes for acceleration of 10 g (sine function with frequency of 200 Hz) are presented. In Figure 3a the spectral leakage is visible. Figure 3b shows the effect of the Hamming windowing function and the leakage is reduced.

**Figure 4:**
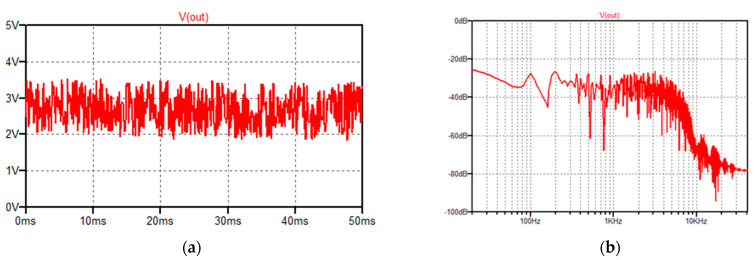
(**a**) The waveform of Gaussian white noise; (**b**) spectral amplitude of the noise, simulation time 500 ms, and *N* = 4096.

The waveform of Gaussian white noise represents machine vibrations (Figure 4a). For a noise amplitude *V_a_* of 1.5 V (peak-to peak), signal-to-noise ratio equal to 5, the amplitude spectrum is depicted in Figure 4b.

## 5. Laboratory Tests of Selected Sensors Exposed to Low Accelerations

**Figure 5:**
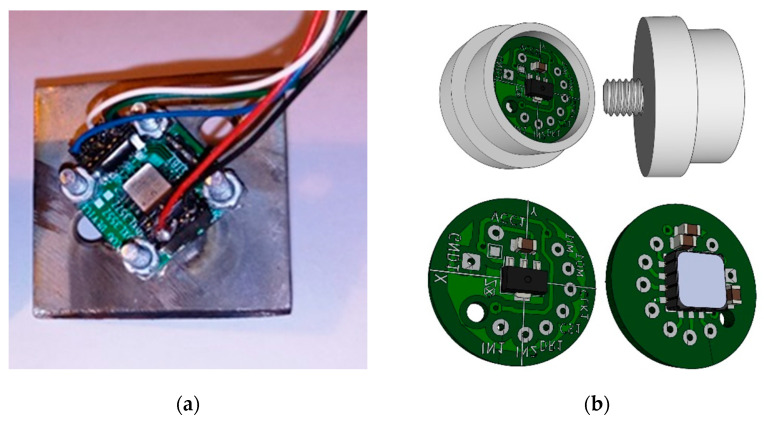
(**a**) ADXL accelerometer on a metal base; (**b**) the designed PCB and the case (stainless steel) with M6 screw.

The aim of the laboratory tests was to determine the static performance characteristics of the selected sensors by a process called static calibration. Three sensors were tested on a shaker. The screw mounting method is not always the most practical, but it is the preferred method for vibration sensors. This type of attachment is considered the most reliable and it is expected to produce the best repeatability of all methods. The ADXL356 accelerometer (Figure 5) was soldered, and the PCB was screwed to the metal base with four M3 screws. The M6 screw was welded to the metal base, which enabled the system to be screwed to a shaker. The sensor was configured for the range of ±10 g, with a sensitivity of 80 mV/g, bandwidth—2.4 kHz and a resonant frequency of 5.5 kHz.

**Figure 6:**
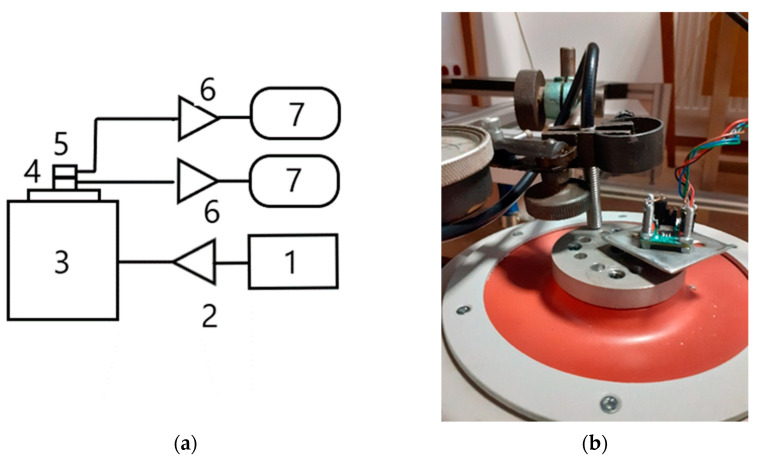
(**a**) Scheme of a vibration test stand: 1—signal generator (Agilent), 2—power amplifier (TIRA), 3—vibration exciter (TIRA), 4—reference sensor FIBER PHILTEC RC62-T2, 5—tested sensor (ADXL356, MEAS 810M- 0025X and TE 820M1-0025), 6—signal amplifiers, 7—RIGOL DM3068 multimeters; (**b**) physical model of a vibration test stand—vibration exciter, reference and tested sensors.

The source of sinusoidal vibrations was a generator connected with the TIRA TV51140 shaker (Figure 6). The sensors (ADXL356, MEAS 810M1-0025X and TE 820M1-0025) were tested using the comparative method in the frequency range of 30–200 Hz. A precise FIBER PHILTEC RC62-T2 laser displacement sensor with a sensitivity of 2.9 mV/µm was used as the reference instrument. The reference sensor was factory calibrated. The selected characteristics of the tested sensors are presented in Table 1.

**Figure 7:**
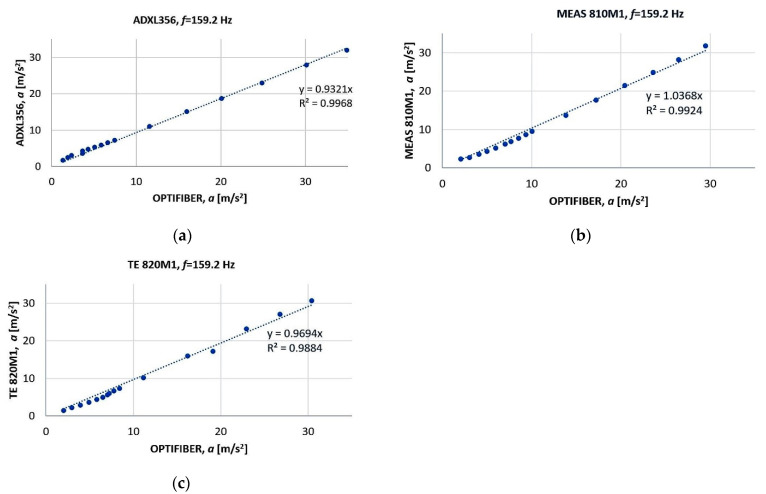
A linear regression line where Y is the acceleration measured by: (**a**) ADXL356 MEMS sensor; (**b**) MEAS 810M1 sensor; (**c**) TE 820M1 sensor, and X is acceleration measured by finding displacement measurement with the use of the FIBER PHILTEC laser sensor.

At the same time, the output voltages of the tested sensor and the reference sensor in the range from 0 to 4 g were recorded. The linear regression models were obtained for the vibration frequency of 159.2 Hz (Figure 7a–c). All sensors were supplied with the voltage of 3.3 V from the Tektronix PS2520G power supply. Experiments were conducted at a temperature of 25 °C.

Other parameters are given in Table 2.

The slope of a linear function *y* = *mx*

(6) $$m=\frac{\sum_{i=1}^{L}x_{i}y_{i}}{\sum_{i=1}^{L}x_{i}^{2}}$$

where *x_i_* is the acceleration determined on the basis of the FIBER PHILTEC sensor and *y_i_* is the acceleration measured by a tested sensor, *L*—number of observations (*L* = 16).

The linear correlation coefficient is described by the equation

(7) $$r=\frac{\sum_{i=1}^{L}x_{i}y_{i}}{\sqrt{\sum_{i=1}^{L}x_{i}^{2}}\sqrt{\sum_{i=1}^{L}y_{i}^{2}}}$$

The standard uncertainty of the coefficient *m* is

(8) $$u\left(m\right)=S_{m}=\frac{m}{r}\sqrt{\frac{1-r^{2}}{L-2}}$$

The coefficient of determination which tells how well the data fit the model

(9) $$R^{2}=\frac{\sum_{i=1}^{L}{\left({\hat{y}}_{i}-\bar{y_{i}}\right)}^{2}}{\sum_{i=1}^{L}{\left(y_{i}-\bar{y_{i}}\right)}^{2}}$$

The residual standard deviation is

(10) $$S_{e}=\sqrt{\frac{\sum_{i=1}^{L}{\left({\hat{y}}_{i}-y_{i}\right)}^{2}}{L-K}}$$

where *K*—number of estimated parameters (*K* = 1).

The coefficient of variation normalizes the volatility by dividing the residual standard deviation by the mean

(11) $$\upsilon =\frac{S_{e}}{{\bar{y}}_{i}}100\%$$

The smallest coefficient of variation and the best linearity were obtained for the ADXL356 sensor. Calculated values are *v* = 9.45% (TE820M1), 6.91% (MEAS810M1) and 5.02% (ADXL356).

## 6. The Design of a Vibration-Based Monitoring System for Hay-Handling Equipment

An inductive sensor of the E2B series (OMRON Corporation, Japan) was used for measuring rotational speed [34]. The sensor is supplied by 10 to 30 VDC. It is a sensor with voltage output PNP and operates in NO (normally open) mode.

**Figure 8:**
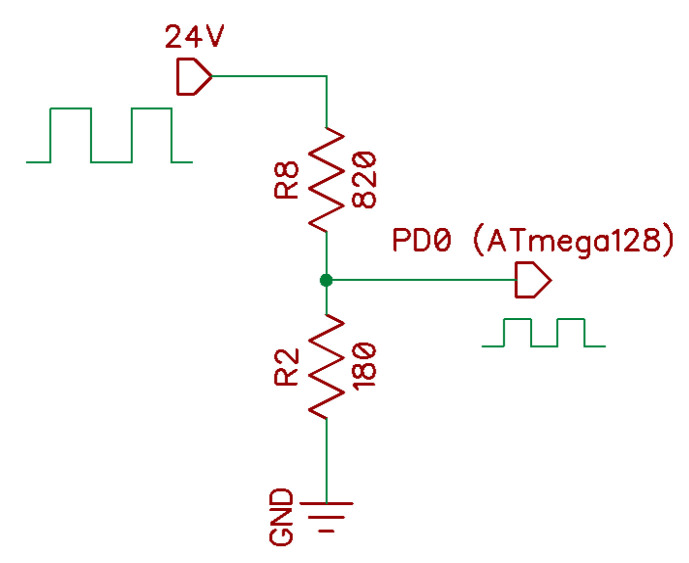
The voltage divider used.

While detecting a metal object within a distance of 10 mm from the sensor’s face, the output is set at 24 DC Volts. Therefore, for measuring pulses coming from the sensor, it is necessary to reduce the voltage by means of a resistive divider to the level acceptable to the microcontroller pin PD0 (Figure 8).

The electronic prototype of the system for recording and analyzing measurement signals was built based on an ATmega128 microcontroller. The software support for on-chip multi-channel ADC (responsible for averaging, decimation, noise reduction) was implemented. A program for calculating selected statistical parameters (such as RMS value, standard deviation and crest factor) of recorded signals was created. Electronic components were reviewed for further development of the hardware layer. The influence of passive-component tolerances on the accuracy of the measured signal was investigated.

**Figure 9:**
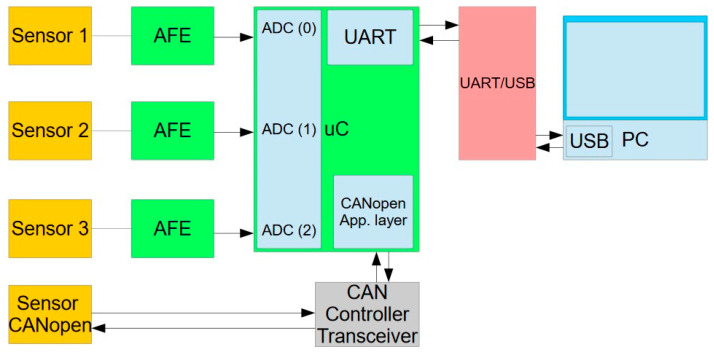
The block scheme of the 4-channel measuring system. AFE—analog front-end (signal conditioning circuitry); uC—Atmega128 microcontroller.

A block diagram of the 4-channel system for processing data from accelerometers and the rotation sensor is presented in Figure 9.

The on-chip ADC operates with a resolution of 10-bits. For precise measurements, the voltage on the AVCC pin was filtered by the LC filter (L = 10 uH, C = 100 nF). The unused ADC pins were blocked. The noise reduction mode was used, which requires the following steps to be performed during a measurement:

1. configuring the ADC in the single conversion mode;
2. disabling the ADC interrupt;
3. executing the sleep () function.

The code that uses ADC Noise Reduction mode consists of three parts.

ISR (ADC_vect)
{
}
void initADC()
{
      ADMUX |= (1<<REFS1)|(1<<REFS0);
      ADCSRA |= (1<<ADEN)|(1<<ADIE);
}
int getADC()
{
      set_sleep_mode(SLEEP_MODE_ADC); //canceller mode
      cli();
      sleep_enable();      //unlock sleep mode
      sei();
      sleep_cpu();       //enter sleep mode
      sleep_disable();     //lock sleep mode
      return ADC;
}

An interrupt service routine (ISR) is necessary because the ADC interrupts must be enabled to prevent the processor from waking up from sleep mode. If they were enabled without this routine, a default routine would be executed, which would reset the microcontroller. Decimation and interpolation were also used in the application to perform measurements via the ADC in the microcontroller.

Contrary to the averaging of the measurement result, the aim of the above-mentioned procedures was to increase the resolution of the sampled signal over the offered 10-bit value. It is known that four signal samples are needed to obtain one extra bit of information. As a result, in order to obtain a 12-bit resolution the values of 16 samples had to be summed and divided by 4. As a result of adding 16 values (10-bit), a 14-bit result is obtained, in which the two youngest bits do not contain any useful information. Doubling the sampling rate will increase the signal-to-noise ratio by 3 dB, while increasing the resolution by 0.5 bit. In general, to increase the resolution by *n* bits, a signal should be sampled at frequency *f_OVS_* [35]

(12) $$f_{OVS}=4\;nf_{s},$$

where *f_s_* is the sampling rate.

At the same time, oversampling reduces the maximum frequency of the sampled signal, which is related to a maximum sampling frequency of the ADC and to a number of additional bits. The frequency limitation can be determined from the formula

(13) $$f_{\max}=\frac{f_{ADC\_\max}}{2\cdot 4n},$$

where *f_ADC_max_* is the maximum sampling rate of the converter. Based on the tests carried out with the ATmega128 microcontroller, the obtained *f_max_* = 62.5 kHz was achieved with an additional 2-bit increase in the ADC resolution.

**Figure 10:**
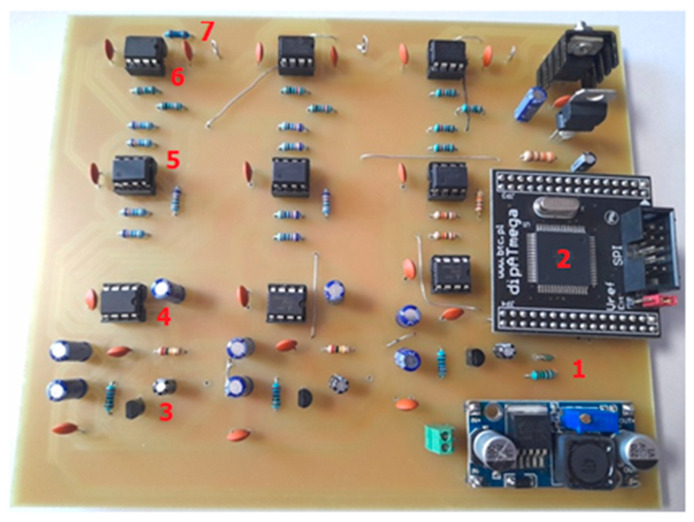
The PCB of the signal converter: 1—voltage divider, 2—ATmega128 microcontroller, 3—LM317 voltage regulator as current limiter, 4—ICL7660 voltage converter, 5—LM833N operational amplifier, 6—MCP6022 dual operational amplifier, 7—anti-aliasing filter RC, LPF corner frequency 5.136 kHz.

In the electronic prototype of the signal converter (Figure 10), a full analog front end for the IEPE sensors (3 channels) and one channel for recording the rotational speed of the machine drive shaft was considered.

The FFT algorithm was implemented in the ATmega128 microcontroller using the C language and assembler. The use of a low-level programming language enabled optimizing calculations in terms of execution time. To this aim, the C language compiler for AVR microcontrollers was not sufficient. Operating directly on internal resources, i.e., working registers, is the most effective way to obtain the smallest code size possible. The calculations were divided into four stages carried out in separate subroutines:

1. creating an array of raw data values to prepare butterfly operations;
2. preparing the Hamming window;
3. performing butterfly operations;
4. arranging the results at the output of a butterfly.

Table 3 presents the most important comparative parameters of the FFT implementation in the microcontroller. The data were obtained as a result of applying a function written in C language in Atmel Studio IDE environment and in AVR assembler.

The computation time of a program written in the AVR assembler is 2–3 times shorter. The FFT computational complexity is defined as a number of multiplication operations.

## 7. Laboratory Tests—Comparison of the Time and Frequency Characteristics of the Constructed Measuring System with the Commercial AVM4000 System

### 7.1. Sinusioidal Excitation

Considering that the system is stable, and its input is sinusoidal *u*(*t*) = *A_u_∙*cos(*ωt*), at a steady state the response is asymptotically sinusoidal *y_ss_*(*t*) = *A_y_∙*cos(*ωt* + *φ*) with the same frequency as the input. The frequency response is

(14) $$H\left(j\omega \right)=\int_{0}^{\infty }h\left(t\right)e^{-j\omega t}dt$$

Then |*H*(*jω*)| gives the amplification factor, i.e.,

(15) $$\left|H\left(j\omega \right)\right|=\frac{RMS\left(y_{ss}\right)}{RMS\left(u\right)}$$

and the phase is a shift between *u* and *y_ss_*.

**Figure 11:**
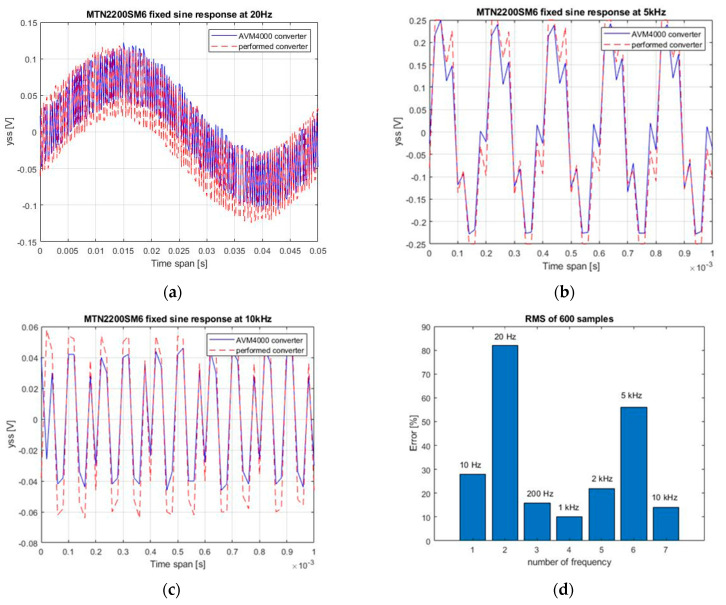
Time response of the tested and commercial AVM4000 converters excited with a function generator: (**a**) sine 20 Hz; (**b**) sine 5 kHz; (**c**) sine 10 kHz; (**d**) absolute relative difference.

The circuit of the designed and commercial AVM4000 converter (together with the MTN2200SM6 sensor) was stimulated with a sinusoidal signal with selected frequencies of 20 Hz (Figure 11a), 5 kHz (Figure 11b) and 10 kHz (Figure 11c) and with an amplitude of 100 mV. An Agilent signal generator was used. Relative differences RMS (*y*_ss_) relating to the input signal amplitude u, presented in Figure 11d, were obtained.

Absolute relative difference related to input amplitude *A_u_* is

(16) $$Error=\frac{\left|\mathrm{RMS}\left(y_{ss\_1}\right)-\mathrm{RMS}\left(y_{ss_{2}}\right)\right|}{A_{u}}100\%$$

The lowest error was observed at 1 kHz. One of the reasons was a phase shift between sine signals up to 100 Hz (Figure 11a). Another reason was the presence of periodic noise in signals at 5 kHz (Figure 11b).

### 7.2. Swept-Sine Frequency Response

**Figure 12:**
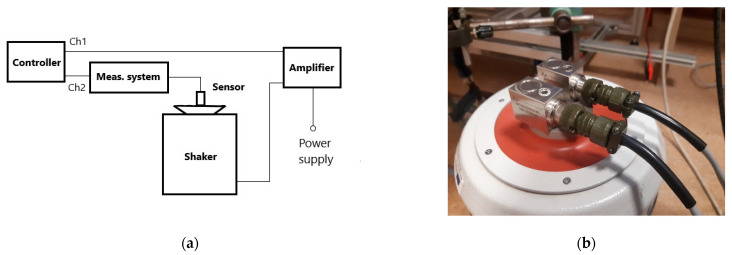
(**a**) Hardware connection diagram for a vibration control test (controller—Agilent Dynamic Signal Analyzer 35670A; meas. system—the performed converter or the AMC Vibro Monitor AVM4000; shaker—TIRA TV51140; amplifier—TIRA BAA1000); (**b**) shaker with mounted MTN2200SM6 sensors.

In order to obtain information about the phase and amplitude differences in the frequency range 10 Hz–10 kHz, a laboratory stand was built, as shown in the diagram (Figure 12a). An Agilent Dynamic Signal Analyzer 35670A was used as the controller. The swept-sine signal excited the TIRA BAA1000 power amplifier and the TIRA TV51140 shaker (Figure 12b). In this way, vibrations of the shaker were induced by a sinusoidal signal with an amplitude of 100 mV.

The normalized input function (Ch1) is a linear sine sweep

(17) $$u\left(t\right)=sin\left(2\pi f_{0}t+\frac{f_{1}-f_{0}}{2T}t^{2}\right),$$

where: *t* is test duration and *f*_0_ and *f*_1_ are starting and ending frequencies, respectively. The measuring system with sensor is a linear, time-variant system with transfer function *H*(*jω*).

**Figure 13:**
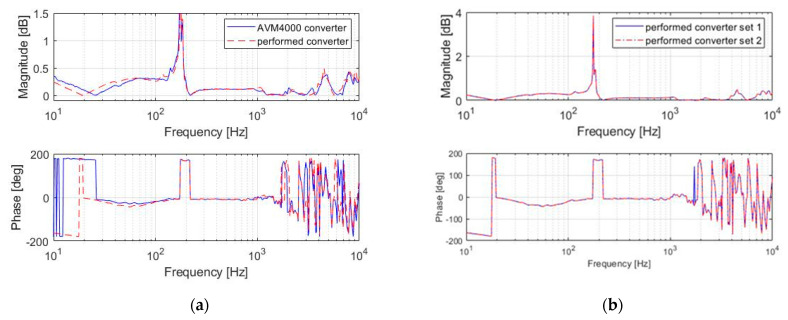
Bode characteristics—amplitude and phase; (**a**)comparison of the designed low-cost system with Sensor 1 and the commercial AVM4000 system with Sensor 2; (**b**) the verification of sensors’ equality by replacing Sensors 1 and 2.

Bode plots of magnitude and phase of transfer function as frequency varied for both (performed and AMV4000) converters are presented in Figure 13a,b

(18) $$Magnitude\;\left[\mathrm{dB}\right]=20\;log_{10}\left|H\left(j\omega \right)\right|,$$

(19) Phase [deg]=arg(H(jω)).

Figure 13a shows the resonance peak, which occurred at a frequency of 180 Hz. The Bode amplitude characteristics of the tested measurement system and the AMV4000 system differed by no more than 15%. The differences in the sign of the phase for both systems were observed up to 30 Hz. The sensors’ equality was verified by replacing Sensors 1 and 2 and connecting to the performed converter. No significant differences were noticed (Figure 13b).

## 8. Machine Vibration Measurements Using the Constructed Measuring System

**Figure 14:**
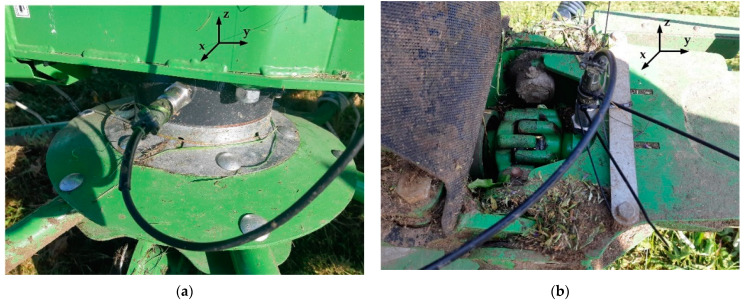
Sensors installed in the rotary tedder P8-890—(**a**) solid grease gearbox (sensor A0, *x*-axis); (**b**) PTO drive shaft bearing (Sensor A1, *z*-axis).

**Figure 15:**
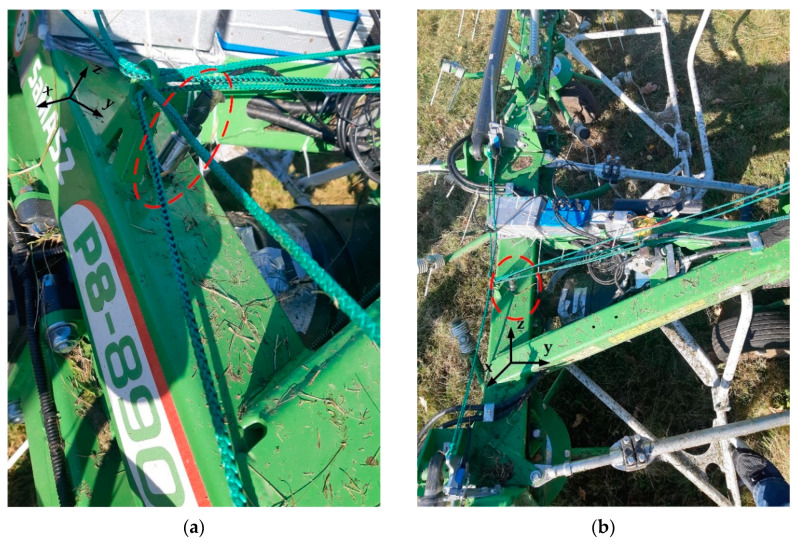
(**a**) Sensor A2 installed in the main frame of the tedder (*z*-axis); (**b**) Sensor A2 and electronic equipment.

Hay handling equipment, such as disk mowers, tedders with a suspension system and trailed rotary tedders, are used in agriculture. Agricultural machinery, such as the rotary tedder P8-890 (SaMASZ, Poland), which supports the process of drying hay by tedding the freshly cut, short-stemmed green plants must be monitored. The deployment of vibration sensors MTN2200SM6 (A0, A1 and A2) is visualized in Figure 14 and Figure 15.

The influence of changes in the tedder’s drive rotational speed on the root-mean square (RMS) accelerations at three characteristic points in the machine was examined. Two tedders P8-890 were tested, the first produced in 2019 (a moderately used machine) and the second produced in 2020 (a more frequently used machine). The aim of this study was to diagnose the condition of the drive shaft bearing, which could provide useful information for improving the durability of such agricultural machinery.

### 8.1. Time-Domain Measurements

**Figure 16:**
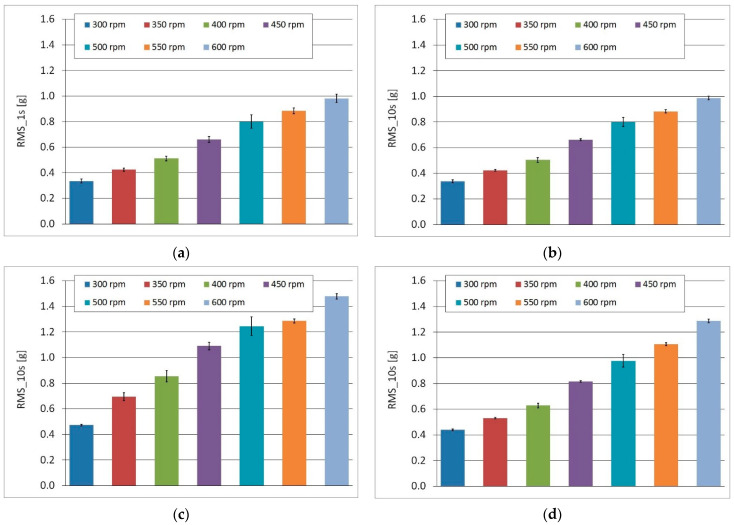
The RMS values of acceleration and error bars (**a**) the RMS acceleration values (sensor A0, x-component) for 1-second signal subsets; (**b**) the RMS acceleration values (sensor A0, x-component) for 10-second signal subsets; (**c**) the RMS acceleration values (Sensor A1, z-component) for 10-second signal subsets; (**d**) the RMS acceleration values (Sensor A2, z-component) for 10-second signal subsets.

The root-mean square (RMS) amplitude values and error bars (standard deviation), which are graphical representations of the variability of data, are presented in Figure 16a–d. The highest RMS amplitudes were observed for the A1 accelerometer. The bar on the graph represents an averaged value of 1-second or 10-second subsets of the full dataset for a selected sensor.

**Figure 17:**
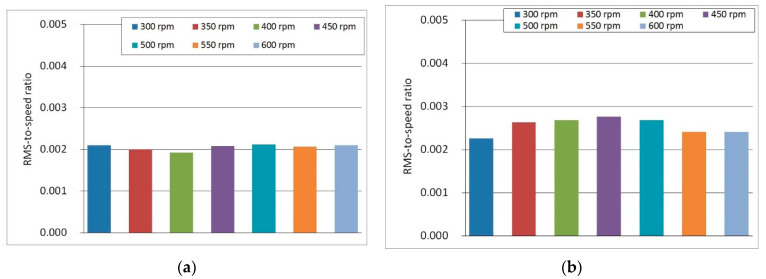
RMS-to-speed ratio experimentally evaluated for the rotary tedder P8-890:(**a**) the main frame of the machine (Sensor A2, *z*-axis); (**b**) PTO drive shaft bearing (Sensor A1, *z*-axis).

The obtained RMS values were converted into new values, to a small extent dependent on the rotational speed of the machine drive, a relationship termed the RMS-to-speed ratio. These values were almost constant within a range of 300–600 rpm. In Figure 17a,b the significant difference between A1 sensor (shaft bearing) and A2 sensor (main frame) is visible. The relative difference in the ratio is about 25% (on average).

In statistics, kurtosis describes the shape of a probability distribution [36]. The datasets with high kurtosis exhibited tail data exceeding the tails of the normal distribution (e.g., five or more standard deviations from the mean). Distributions with low kurtosis exhibited tail data that were generally less extreme than the tails of the normal distribution. The values of the RMS, the RMS-to-speed ratio and kurtosis are presented in Table 4. The RMS value rose with rotational speed; however, kurtosis was almost constant for sensors A0 (gearbox) and A2 (frame). For Sensor A1 (bearing), kurtosis was more variable, being highest at 400 rpm.

**Figure 18:**
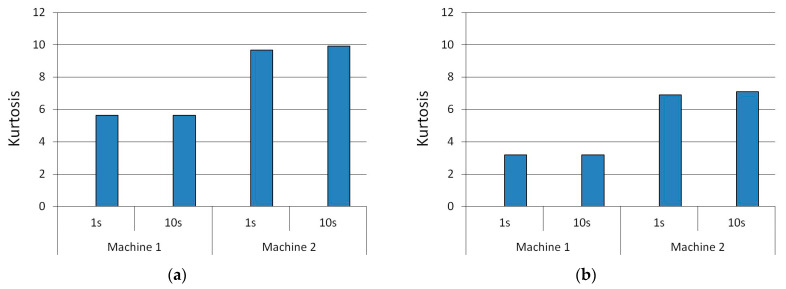
The values of kurtosis for 1-second and 10-second signal subsets at 400 rpm (**a**) Sensor A1; (**b**) Sensor A2.

The values of kurtosis of the shaft bearing vibrations in a function of changes in the rotational speed of the drive shaft at two different points (Sensors A1 and A2) of the machine were evaluated and are presented in Figure 18. The bars on the graph correspond to the averaged values of 1-second or 10-second subsets of the full dataset for Sensors A1 and A2 as shown in Table 5. As can be observed, the length of subset has almost no influence on the kurtosis value.

The normal distribution was found to have a kurtosis of three. A kurtosis higher than three is known as a leptokurtic distribution. Kurtosis values higher than 3 indicate that the distribution has more outliers falling relatively far from the mean. As shown in Figure 18, the mean kurtosis values of the machines differed significantly at both points in the machine. Values closer to 3 were observed for Machine 1. Figure 18a shows that the bearing in Machine 2 could be classified as more worn out than that in Machine 1. This is also confirmed by an analysis of the crest factor (Table 6). The crest factor is the peak amplitude of the vibration waveform divided by the RMS value of the waveform.

### 8.2. Spectral Analysis

**Figure 19:**
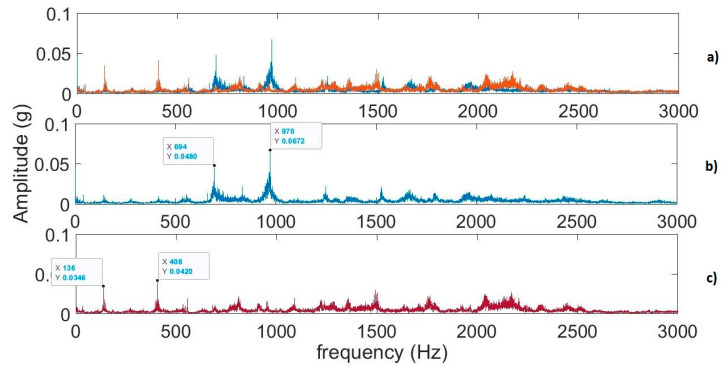
The RMS amplitude spectra of signals from sensor A0 (*x*-axis) at rotational speed 550 rpm: (**a**) both machines; (**b**) Machine 1; (**c**) Machine 2.

The FFT and techniques in the frequency domain can be used for more detailed information about health condition. When performing rotating machinery diagnostics, certain frequency components relate to specific mechanical parts within the machine. In the RMS amplitude spectrum, the components from shafts and gears are in a much lower frequency band, i.e., typically from a few hertz to 3 kHz. They have much more energy than pulses from bearings. The RMS amplitude spectra of the solid grease gearbox (sensor A0) in two rotary tedders are presented in Figure 19 (sampling frequency 10 kHz).

In order to detect changes that occurred in the faulty gearbox (Machine 2) in relation to the intact gearbox, it was possible to determine RMS or energy values within the selected bands of 130–150 Hz and of 400–420 Hz, provided that the above-mentioned parameters at the frequencies 694 Hz and 970 Hz dropped significantly. Computations of the RMS values in the selected bands were enabled to save processing time.

## 9. Conclusions

This article focuses on the design of an electronic prototype for a low-cost vibration-based machine monitoring system. So far, simulation studies have been carried out on adjusting the sensor to the measurement system (Figure 1 and Figure 2). A 4-channel signal converter based on AVR microcontroller ATmega128 with a 10-bit ADC was designed. The ADC in our instrument operated on a maximal sampling frequency *f_max_* = 62.5 kHz, and an additional 2-bit increase in ADC resolution was achieved. The most important comparative parameters of the FFT implementation showed that ATmega128 with implemented software written in the assembler was more efficient than in the C language. The computation times were 2–3 times shorter. However, due to hardware limitations, the frequency resolution was low. It is about 10 Hz for a bandwidth of 10 kHz.

The system operated with piezoelectric sensors. During Phase 3 of the prototyping, several low-cost sensors were tested in the laboratory. The most reliable experimental results and the best linearity were obtained with the ADXL356 sensor. Better metrological parameters (Table 2) of static characteristics were obtained in the range of small acceleration amplitudes (up to 4 g) with this sensor as compared to the TE820M1 and MEAS810M1 sensors. However, its frequency bandwidth was smaller, which limited its use for bearing testing.

The results of measurements on a real hay-handling machine confirmed the capability of the online monitoring system for a simple diagnostic. The linear dependence of RMS values of acceleration on rotational speed is observable in Figure 16. The research proved that one-second subsets are sufficient for processing signals in a time domain (Figure 18). The RMS-to-speed ratio had a different value at each analyzed point in the machine (Table 4). For a more advanced diagnostic of machine components, the use of frequency techniques is needed. Due to the long computation time and low frequency resolution, in this study, it was necessary to calculate RMS values only in the selected bands (Figure 19).

The proposed measuring system can be used for improving the durability of agricultural machinery, and it is under development. Currently, it is used for monitoring two elements in a tedder (gearbox, shaft bearing). However, more advanced methods in the frequency domain than those performed in this paper must be implemented in the microcontroller. One such method is spectral kurtosis (SK), which enables the graphing of a kurtogram and a frequency spectrum [37]. For bearing fault diagnosis, the sampling rate must be higher, as well as the spectral resolution of the constructed device (Table 3). However, the time-domain statistics used in this study allowed assessment of the significant differences in vibrations of the shaft bearing and the gearbox of the two machines (Table 5 and Table 6). The key benefit of such a system is that it is possible to predict potential problems and plan maintenance in advance.

In the framework of a project, the plan is to construct a condition monitoring system for early fault detection in agricultural machinery. This can provide deeper machine analytics of condition monitoring in order to diagnose problems. It is important in the context of avoiding unplanned downtime as well as reducing high costs through excessive planned maintenance. A remote machine monitoring system that works via smartphones is currently under development. This system will send warnings to farmers in the form of short messages, and will help improve the overall efficiency and effectiveness of agricultural operations.

## Figures and Tables

**Table 1:** The selected characteristics of the tested sensors.

Sensor	Sensitivity [mV/g]	Range [g]	Bandwidth [Hz]	Spectral Noise at 100 Hz [mg/√Hz]
TE 820M1	50 (±30%)	±25	2–10,000	0.070
MEAS 810M1	50 (±30%)	±25	2–6000	0.040
ADXL356	80	±10	2400	0.075

**Table 2:** Linear regression parameters *y* = *mx*. *u*(*m*)—standard uncertainty of the slope *m*; *R*^2^—the coefficient of determination; *S_e_*—the residual standard deviation.

Sensor	*m*	*u*(*m*)	*R* ^2^	*S_e_*
TE 820M1	0.9694	±0.0175	0.9951	1.0016
MEAS 810M1	1.0368	±0.0142	0.9972	0.8408
ADXL356	0.9321	±0.0089	0.9986	0.5421

**Table 3:** The most important comparative parameters of the FFT implementation in ATmega128.

	FFT Computation Time [ms]			
Signal Sample Size *N*	AVR C Compiler	AVR Assembler	FFT Frequency Resolution [Hz]	FFT Computational Complexity
32	4.63	1.44	312.5	80
64	8.86	3.75	156.25	192
128	17.72	7.82	78.12	448
256	35.91	15.44	39.06	1024
512	73.22	31.25	19.53	2304
1024	156.30	62.83	9.76	5120

**Table 4:** The RMS amplitude values, the RMS-to-speed ratio and kurtosis. Averaged values for 1-second signal subsets in the full dataset from 3 sensors installed in Machine 1.

	Sensor A0, *x*-Axis			Sensor A1, *z*-Axis			Sensor A2, *z*-Axis		
Rotational Speed *ω* [rpm]	RMS [g]	RMS-to Speed Ratio	Kurtosis 1 s	RMS [g]	RMS-to Speed Ratio	Kurtosis 1 s	RMS [g]	RMS-to Speed Ratio	Kurtosis 1 s
300	0.3360	0.0016	3.7123	0.4727	0.0023	4.5151	0.4397	0.0021	3.2384
400	0.5125	0.0016	3.5629	0.8788	0.0027	5.6528	0.6399	0.0019	3.1866
500	0.8010	0.0017	3.6982	1.2381	0.0027	4.2937	0.9798	0.0021	3.2687
550	0.8856	0.0017	3.3869	1.2908	0.0024	3.7887	1.1106	0.0021	3.1356
600	0.9816	0.0016	3.2818	1.4740	0.0024	3.8324	1.2834	0.0021	3.0822

**Table 5:** The values of kurtosis (KU). Averaged values for 1-second and 10-second signal subsets in the whole dataset from sensors installed in two machines.

	Machine 1				Machine 2			
	Sensor A1, *z*-Axis		Sensor A2, *z*-Axis		Sensor A1, *z*-Axis		Sensor A2, *z*-Axis	
Rotational Speed *ω* [rpm]	KU 1 s	KU 10 s	KU 1 s	KU 10 s	KU 1 s	KU 10 s	KU 1 s	KU 10 s
300	4.5151	4.5818	3.2384	3.2557	-	-	-	-
400	5.6528	5.6463	3.1866	3.1953	9.6731	9.9327	6.9006	7.1002
500	4.2937	4.2811	3.2687	3.2695	-	-	-	-
550	3.7887	3.7870	3.1356	3.1335	-	-	-	-
600	3.8324	3.8476	3.0822	3.0854	-	-	-	-

**Table 6:** The values of the crest factor (CF): averaged values for 1-second and 10-second signal subsets in the whole dataset from sensors installed in two machines.

	Machine 1				Machine 2			
	Sensor A1, *z*-Axis		Sensor A2, *z*-Axis		Sensor A1, *z*-Axis		Sensor A2, *z*-Axis	
Rotational Speed *ω* [rpm]	CF 1 s	CF 10 s	CF 1 s	CF 10 s	CF 1 s	CF 10 s	CF 1 s	CF 10 s
400	5.9640	6.1619	4.2333	4.2991	9.5097	10.0555	7.2260	7.5820

## Data Availability

Not applicable.
